# Confirmed SARS-CoV-2 infection in Scottish neonates 2020–2022: a national, population-based cohort study

**DOI:** 10.1136/archdischild-2022-324713

**Published:** 2023-01-06

**Authors:** Anna Goulding, Fiona McQuaid, Laura Lindsay, Utkarsh Agrawal, Bonnie Auyeung, Clara Calvert, Jade Carruthers, Cheryl Denny, Jack Donaghy, Sam Hillman, Lisa Hopcroft, Leanne Hopkins, Colin McCowan, Terry McLaughlin, Emily Moore, Lewis Ritchie, Colin R Simpson, Bob Taylor, Lynda Fenton, Louisa Pollock, Chris Gale, Jennifer J Kurinczuk, Chris Robertson, Aziz Sheikh, Sarah Stock, Rachael Wood

**Affiliations:** 1 Public Health Scotland, Edinburgh, UK; 2 Department of Child Life and Health, University of Edinburgh, Edinburgh, UK; 3 School of Medicine, University of St Andrews, St Andrews, UK; 4 School of Philosophy, Psychology and Language Sciences, The University of Edinburgh, Edinburgh, UK; 5 Usher Institute, University of Edinburgh, Edinburgh, UK; 6 Nuffield Department of Primary Care Health Sciences, University of Oxford, Oxford, UK; 7 Academic Primary Care, University of Aberdeen, Aberdeen, UK; 8 School of Health, Victoria University of Wellington, Wellington, New Zealand; 9 Child Health, University of Glasgow, Glasgow, UK; 10 Department of Paediatric Infectious Diseases and Immunology, Royal Hospital for Children, Glasgow, UK; 11 Academic Neonatal Medicine, Imperial College London, London, UK; 12 National Perinatal Epidemiology Unit, Oxford University, Oxford, UK; 13 Department of Mathematics and Statistics, University of Strathclyde, Glasgow, UK; 14 Obstetrics and Gynaecology, MRC Centre for Reproductive Health University of Edinburgh, Edinburgh, UK

**Keywords:** COVID-19, neonatology, epidemiology

## Abstract

**Objectives:**

To examine neonates in Scotland aged 0–27 days with SARS-CoV-2 infection confirmed by viral testing; the risk of confirmed neonatal infection by maternal and infant characteristics; and hospital admissions associated with confirmed neonatal infections.

**Design:**

Population-based cohort study.

**Setting and population:**

All live births in Scotland, 1 March 2020–31 January 2022.

**Results:**

There were 141 neonates with confirmed SARS-CoV-2 infection over the study period, giving an overall infection rate of 153 per 100 000 live births (141/92 009, 0.15%). Among infants born to women with confirmed infection around the time of birth, the confirmed neonatal infection rate was 1812 per 100 000 live births (15/828, 1.8%). Two-thirds (92/141, 65.2%) of neonates with confirmed infection had an associated admission to neonatal or (more commonly) paediatric care. Six of these babies (6/92, 6.5%) were admitted to neonatal and/or paediatric intensive care; however, none of these six had COVID-19 recorded as their main diagnosis. There were no neonatal deaths among babies with confirmed infection.

**Implications and relevance:**

Confirmed neonatal SARS-CoV-2 infection was uncommon over the first 23 months of the pandemic in Scotland. Secular trends in the neonatal confirmed infection rate broadly followed those seen in the general population, although at a lower level. Maternal confirmed infection at birth was associated with an increased risk of neonatal confirmed infection. Two-thirds of neonates with confirmed infection had an associated admission to hospital, with resulting implications for the baby, family and services, although their outcomes were generally good. Ascertainment of confirmed infection depends on the extent of testing, and this is likely to have varied over time and between groups: the extent of unconfirmed infection is inevitably unknown.

**WHAT IS ALREADY KNOWN ON THIS TOPIC**

- SARS-CoV-2 infection in neonates appears uncommon, but some studies have suggested that neonates are at higher risk than older children of severe infection.
- Population-based data on neonates with confirmed infection are lacking: most studies to date have only included babies of infected mothers or those admitted to the hospital.

**WHAT THIS STUDY ADDS**

- Confirmed SARS-CoV-2 infection in all neonates in Scotland from 1 March 2020 to 31 January 2022 was uncommon, occurring in 0.15% (141/92 009) of live births.
- Confirmed maternal SARS-CoV-2 infection around the time of birth was associated with an increased risk of confirmed neonatal infection at 1.8% (15/828) of live births.
- 65.2% (92/141) of neonates with confirmed infection had an associated hospital admission with 6.5% (6/92) involving neonatal/paediatric intensive care: there were no neonatal deaths.

**HOW THIS STUDY MIGHT AFFECT RESEARCH, PRACTICE OR POLICY**

- Ascertainment of confirmed infection depends on the extent of testing, and this is likely to have varied over time and between groups.
- Continued data collection and vigilance will be important to assess the ongoing impact of SARS-CoV-2 in the neonatal population as the pandemic evolves.

## Introduction

Confirmed neonatal infection with SARS-CoV-2, defined as a positive viral test in the first 27 days after birth, is uncommon.^1–4^ A UK study identified 66 neonates with confirmed infection admitted to the hospital between 1 March and 30 April 2020, giving an estimated infection/admission rate of 5.6/10 000 live births.^1^ Less than 2% of babies born to women with confirmed infection around the time of birth develop confirmed infection themselves.^5^ However, babies born to women with confirmed infection are more likely to be born prematurely or be admitted to the neonatal unit, regardless of infant SARS-CoV-2 status.^5–8^ Neonates with confirmed infection can develop severe disease; however, reports on the proportion of SARS-CoV-2 positive neonates requiring admission to intensive care vary, depending on the definition of intensive care.^1 2 9 10^

To date, most neonatal SARS-CoV-2 studies have focused on the risk and consequences of transmission to the neonate from an infected mother.^5 11^ However, in the neonatal period, babies are exposed to multiple other potential sources of infection, for example, other caregivers and healthcare professionals. Previous studies have included neonates admitted to the hospital with a positive SARS-CoV-2 test^1^; however, population-level data including those testing positive in the community are lacking.^4^ The aim of this study was to examine all confirmed cases of SARS-CoV-2 infection in infants aged 0–27 days in Scotland from 1 March 2020 to 31 January 2022.

## Methods

### Study population

Detailed methods are provided as online supplemental material. In brief, data were obtained from the ‘COVID-19 in Pregnancy in Scotland’ (COPS) study dataset.^12 13^ COPS contains data on all ongoing and completed pregnancies to women in Scotland, and liveborn babies resulting from those pregnancies, from 1 January 2015 onwards linked to information on SARS-CoV-2 viral testing, admissions to neonatal and paediatric care, and deaths.^12^ For this study, we included all live births in Scotland between 1 March 2020 and 31 January 2022 with a valid Community Health Index (CHI) number available within the COPS dataset.

10.1136/fetalneonatal-2022-324713.supp1 Supplementary data

### Identifying confirmed SARS-CoV-2 infections

COPS includes information on all positive SARS-CoV-2 viral tests undertaken on women and babies within the cohort.^12 14^ Up to and including 5 January 2022, confirmed SARS-CoV-2 infection was defined as a positive viral reverse transcription–polymerase chain reaction (RT-PCR) test result. From 6 January 2022 onwards, confirmed infection was defined as a positive viral RT-PCR or a positive lateral flow device (LFD) test (unless the positive LFD result was followed by a negative RT-PCR result within 48 hours). For any individual, the date that their first positive test sample was taken was used as the date of onset of their first episode of infection. Confirmed neonatal infection was defined as a positive test with date of onset from birth to 27 days old inclusive. Maternal infection at the time of birth was defined as a confirmed infection with date of onset in the 14 days leading to birth, on the day of birth or the day after giving birth.

For all babies with confirmed neonatal infection, data were obtained from the COPS database regarding the age of the baby in days at date of onset of infection; maternal age, socioeconomic level, ethnicity, and infection status at the time of birth: and the baby’s sex and gestation at birth. Maternal socioeconomic level was based on the Scottish Index of Multiple Deprivation quintile.^15^

### Identifying hospital admissions associated with confirmed neonatal SARS-CoV-2 infection

A hospital admission associated with confirmed neonatal SARS-CoV-2 infection was defined as an admission of a baby with confirmed neonatal infection to neonatal or paediatric care (1) where the date of onset of infection was in the 7 days prior to, or during, the admission (hence date of admission at up to 27+7=34 days old inclusive), or (2) where the admission occurred at any point in the neonatal period (hence date of admission at up to 27 days old inclusive) if COVID-19 was recorded as the main diagnosis (International Classification of Diseases, 10th Revision, code U07.1 or U07.2). An ‘admission’ was defined as an entire hospital stay from admission to discharge. The main diagnosis was taken from the first episode of care during an admission.^16^

Admissions to neonatal units were identified through the Scottish Birth Record^17^ and admissions to paediatric wards through hospital inpatient and day-case discharge records (SMR01^18^). SARS-CoV-2-associated admission records were analysed to identify the highest level of care provided in the neonatal unit or whether they included an episode in a paediatric intensive care unit (PICU) (‘significant facility’ coded to 13^19^), length of stay, whether COVID-19 was listed as the main diagnosis and whether the infection was likely to be nosocomial. A probable nosocomial infection was defined as when the first positive viral test was taken on day 7 or later of an ongoing admission.

### Calculation of rates and CIs

All data reported here are descriptive only with no formal statistical comparisons. Rates were calculated using the number of babies with confirmed neonatal infection and the total number of live births during the study time period. The CIs were calculated using Wilson score estimates. The analysis and generation of figures were carried out using R V.3.6.1 and RStudio V.1.1.463, and codes are available online (https://github.com/Public-Health-Scotland/COPS-public.git).

## Results

Overall, 92 032 live births in Scotland between 1 March 2020 and 31 January 2022 were included in the COPS dataset, of whom 92 009 had a valid CHI number. One-hundred and forty-two neonates with confirmed SARS-CoV-2 were identified from the national viral testing data. Of these, 141 neonates were within the COPS cohort and were included in the analysis. The remaining baby was presumed to have been born outside of Scotland and was excluded.

### Neonatal infection rates

**Figure 1:**
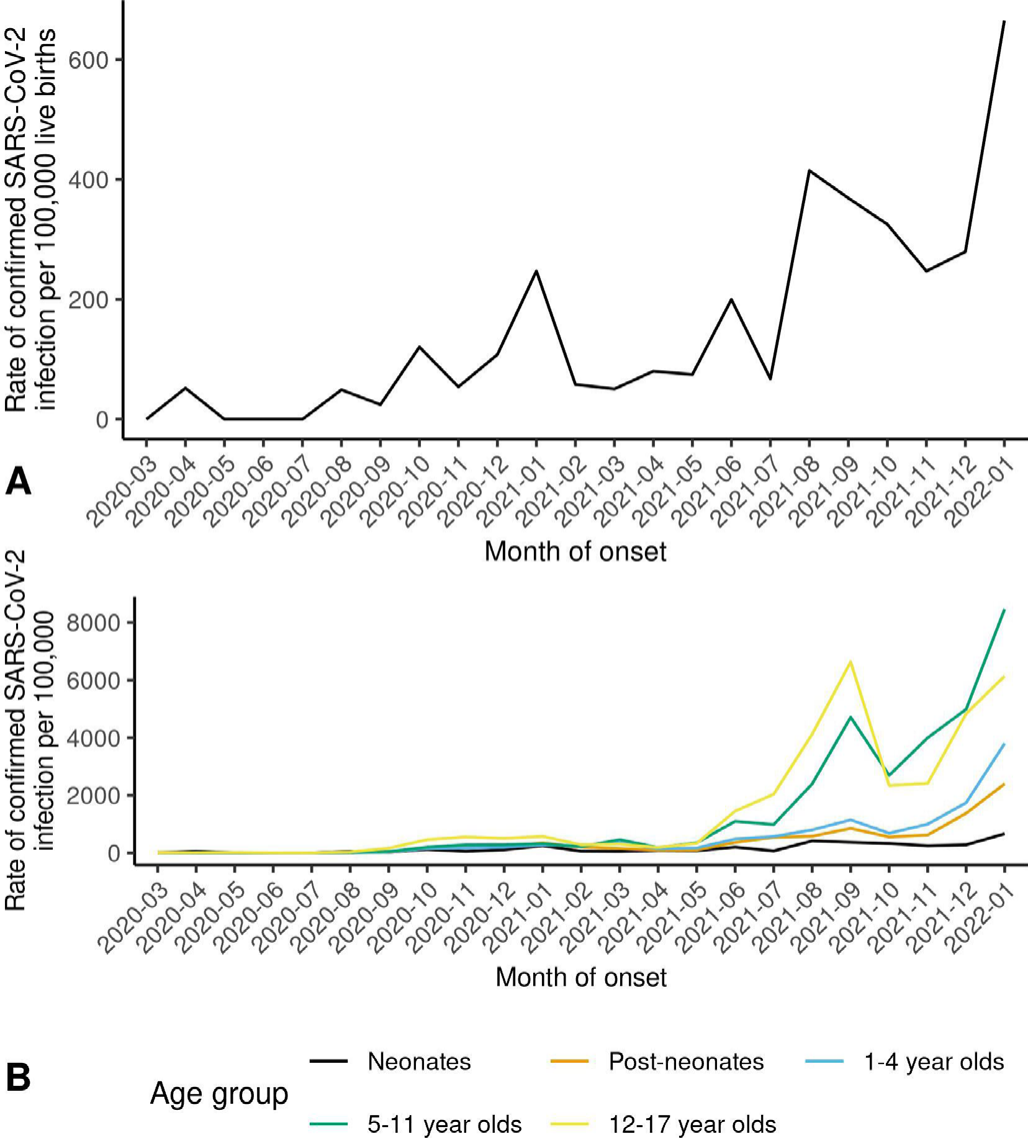
Scotland, March 2020–January 2022. (A) Monthly rate of confirmed SARS-CoV-2 infection in neonates (babies aged 0–27 days) per 100 000 live births. (B) Monthly rates of confirmed SARS-CoV-2 infection in neonates, postneonates (babies aged 28–364 days) and children aged 1–4 years, 5–11 years and 12–17 years per 100 000 live births (neonates) or population (older age groups). For further details on data sources and estimates, see online supplemental table S2.

Across the study period, the overall neonatal confirmed SARS-CoV-2 infection rate was 153 per 100 000 live births; however, this varied by month from 0 to 665 per 100 000 live births (figure 1A and online supplemental table S1). For context, figure 1B shows the neonatal confirmed infection rate alongside the rates for older children (drawing on other population-based data held by Public Health Scotland) (online supplemental table S2). The neonatal infection rate was consistently the lowest, though all paediatric age groups showed similar peaks of infection in autumn 2021 and December 2021/January 2022. The monthly rates of confirmed infection in pregnant women are presented in online supplemental table S3.

### Infant and maternal characteristics and confirmed neonatal SARS-CoV-2 infection

The infant and maternal characteristics of neonates with confirmed SARS-CoV-2 infection are shown in tables 1 and 2. Rates of confirmed neonatal infection were highest among babies born to younger women and to women from more deprived areas, although CIs overlapped. Rates of confirmed neonatal infection among babies born to women from minority ethnic groups were uncertain due to low numbers. The rate of confirmed neonatal infection was substantially higher in babies born to women with (compared with without) confirmed infection at the time of birth; however, the absolute risk of confirmed neonatal infection was low in both groups (table 2).

**Table 1:** Infant characteristics and confirmed neonatal SARS-CoV-2 infection

	Total live births (n)	Neonates SARS-CoV-2 positive (n)	Rate per 100 000 live births	Lower CI	Upper CI
Infant sex					
Male	47 232	79	167	133	210
Female	44 777	62	138	107	179
Gestation at birth					
Preterm (22–36 weeks)	7231	17	235	142	385
Earlier preterm (22–33 weeks)	1946	3	154	40	490
Later preterm (34–36 weeks)	5285	14	265	151	456
Term+ (37–44 weeks)	84 735	124	146	122	175
Unknown	43	0	–	–	–
Total	92 009	141	153	129	181

**Table 2:** Maternal characteristics and confirmed neonatal SARS-CoV-2 infection

	Total live births (n)	Neonates SARS-CoV-2 positive (n)	Rate per 100 000 live births	Lower CI	Upper CI
Maternal age (years)					
≤19	3248	12	369	200	664
20–24	12 886	26	202	135	300
25–29	26 723	41	153	111	210
30–34	30 837	47	152	113	204
35–39	15 471	12	78	42	140
≥40	2744	3	109	28	348
Unknown	100	0	–	–	–
Maternal deprivation level (SIMD quintile)					
1—most deprived	21 189	47	222	165	298
2	18 501	29	157	107	228
3	16 860	21	125	79	194
4	19 374	26	134	90	200
5—least deprived	16 018	18	112	69	181
Unknown	67	0	–	–	–
Maternal ethnicity					
White	77 481	118	152	127	183
South Asian	3073	8	260	121	534
Black/Caribbean/African	1431	3	210	54	666
Mixed or other ethnic group	3222	5	155	57	384
Unknown	6802	7	103	45	222
Maternal SARS-CoV-2 infection status at birth					
Confirmed SARS-CoV-2 infection	828	15	1812	1055	3042
No confirmed SARS-CoV-2 infection	91 181	126	138	116	165
Total	92 009	141	153	129	181

SIMD, Scottish Index of Multiple Deprivation.

### Age in days at date of first positive test

**Figure 2:**
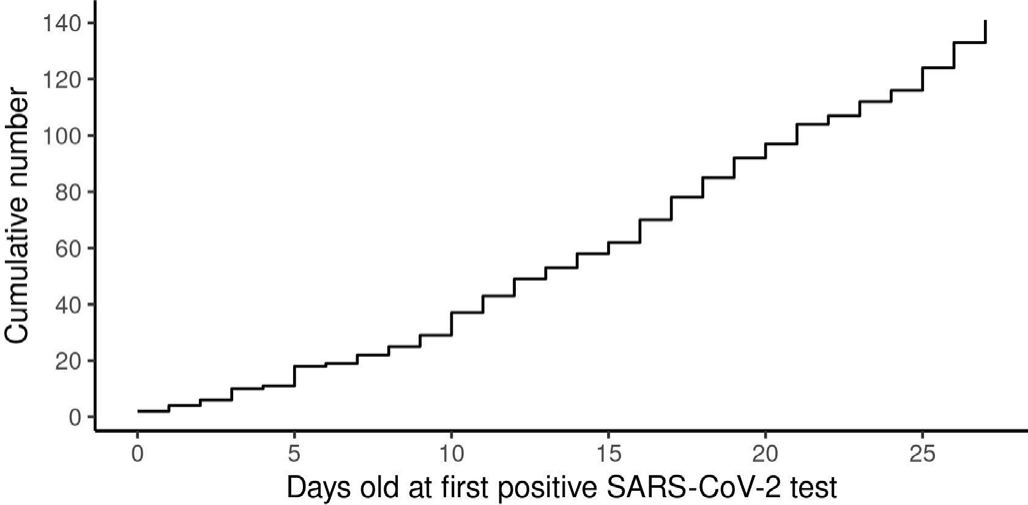
Cumulative number of confirmed SARS-CoV-2 infections in neonates by age at time of first positive test.

The incidence of confirmed infection over the neonatal period followed a linear trend (figure 2 and online supplemental table S4). Of the 15 babies with confirmed neonatal infection who were born to a woman with confirmed infection at birth, none tested positive at <2 days of age, 9 first tested positive between days 2 and 7, and 6 on day 8 or later. Thus, none of these babies met the WHO criteria for confirmed or possible in utero or intrapartum transmission, and would be classified as ‘indeterminate’ status for early postnatal SARS-CoV-2 maternal to child transmission.^11^

### Hospital admission and outcomes of babies with confirmed neonatal SARS-CoV-2 infection

Of the 141 babies with confirmed neonatal infection, 92 (92/141, 65.2%) had a total of 101 admissions to neonatal and/or paediatric care that were temporally associated with their positive SARS-CoV-2 test (first positive test taken in the 7 days prior to, or during, the admission). There were no additional associated admissions in the neonatal period with COVID-19 coded as the main diagnosis that did not meet the temporal association criteria.

None of the six SARS-CoV-2-associated admissions to a neonatal unit had COVID-19 coded as the main diagnosis, and three involved probable nosocomial infection. By contrast, 66% (64/97) of the SARS-CoV-2-associated admissions to paediatric care had COVID-19 coded as the main diagnosis, and only one involved probable nosocomial infection. Six of the babies with an associated admission (6/92, 6.5%) had a total of six admissions involving an episode of care in neonatal and/or paediatric intensive care (with two involving a transfer between neonatal and paediatric intensive care). None of these admissions had COVID-19 coded as the main diagnosis (table 3). Over the 23-month study period, the proportion of babies with confirmed neonatal infection that were admitted to hospital remained broadly consistent (online supplemental table S5 and online supplemental figure S1).

**Table 3:** Hospital admissions temporally associated with a positive SARS-CoV-2 test among babies with confirmed neonatal infection

	COVID-19 coded as main diagnosis		All
	Yes	No	
Admissions to neonatal unit (n=6)			
Maximum level of care			
Intensive care	0	2	2
High dependency or special care only	0	4	4
Probable nosocomial infection			
Yes	0	3	3
No	0	3	3
Total	0	6	6
Mean LOS in days (median, lower–upper quartile)	NA	28.8 (22.5, 17.0–34.8)	28.8 (22.5, 17.0–34.8)
Admissions/transfers to paediatric care (n=97)			
Maximum level of care			
PICU	0	6	6
No PICU	64	27	91
Probable nosocomial infection			
Yes	0	1	1
No	64	32	96
Total	64	33	97
Mean LOS in days (median, lower–upper quartile)	1.6 (1, 0.8–2.3)	4.1 (2, 1–3)	2.4 (1, 1–3)

There were 101 separate admissions to neonatal or paediatric care of 92 neonates (two admissions involved a transfer from neonatal to paediatric intensive care).

PICU, paediatric intensive care unit; LOS, length of stay; NA, not applicable.

There were no neonatal deaths among the 141 babies with confirmed neonatal infection. The background neonatal mortality rate in March 2020–January 2022 was 2.2/1000 live births (206/91 864, 95% CI 2.0 to 2.6) among uninfected babies.

## Discussion

These results show that confirmed neonatal SARS-CoV-2 infection was uncommon in Scotland over the first 23 months of the pandemic, with only 141 neonates having confirmed infection between 1 March 2020 and 31 January 2022. The secular trend in the confirmed neonatal infection rate followed that seen in older age groups, although at much lower levels. Factors associated with higher infection rates among pregnant women, such as young maternal age and living in a more deprived area, were associated with higher neonatal infection rates. The rate of confirmed neonatal infection was significantly higher in babies born to women with (compared with without) confirmed infection at the time of birth. Two-thirds of neonates with confirmed SARS-CoV-2 infection had an associated hospital admission, primarily to paediatric care. However, only 6.5% of admitted babies required intensive care, and none of these babies had COVID-19 coded as their main diagnosis. There were no neonatal deaths among babies with confirmed infection.

These data align with previous studies on admission rates for neonatal SARS-CoV-2^1 4^ and give an insight into the total burden of confirmed neonatal infection in the UK. A strength of this study is that it takes a population-level view of neonatal SARS-CoV-2 infection, rather than confining results to only those born to infected women, or only those admitted to hospital. Our data also encompass almost 2 years of the pandemic, including the emergence of new viral variants and the introduction of COVID-19 vaccines. In keeping with published rates,^5^ we found just under 2% of babies born to women with confirmed infection at the time of birth had confirmed neonatal infection. Not all babies of infected women were tested (see further), and rates may have been higher if all were screened.^20^

This was an observational study, with cases of confirmed infection identified through the results of ‘real-world’ testing carried out in the community and hospitals across Scotland. Not all babies will have been tested, and some infections may therefore have been missed. The extent of unconfirmed infection is inevitably unknown. The proportion of all infections that are confirmed will be influenced by the extent of testing, and this is likely to have varied over time and between groups.

During the study period, infants may have been tested due to having clinical signs of infection, having contact with a case, or as part of routine hospital admission testing.^21^ Around half of neonates with SARS-CoV-2 infection appear well or show mild clinical signs which may not prompt testing.^1 2 10 22–25^ Scottish policy recommended testing of all emergency hospital admissions (including to paediatric and maternity care) from early December 2020.^26^ Guidance on testing of neonates varies between countries.^22^ In the UK, professional guidance on testing in neonatal care in place throughout our study period recommended testing babies born to mothers with confirmed infection who required admission, those readmitted from the community, those with clinically suspected COVID-19 and weekly testing for those receiving respiratory support. Testing neonates less than 72 hours old was not recommended due to difficulties interpreting results. Routine testing of newborns, including those born to mothers with confirmed infection, who were well and remained in postnatal settings was not recommended.^27^ Access to community-based RT-PCR testing became widely available (including for children) from August 2020 and free home LFD testing was available from April 2021.

It is likely that over the study period, babies showing more severe clinical signs, those born to mothers with confirmed infection and those admitted to neonatal or paediatric care (for whatever reason) are more likely to have undergone testing, and hence ascertainment of confirmed infection is likely to have been more complete in these groups. However, this is unlikely to account for the greater than 10-fold increased risk of confirmed neonatal infection seen in babies born to mothers with confirmed infection at the time of birth.

Reassuringly, we demonstrate that the clinical outcomes of neonates with confirmed SARS-CoV-2 infection are good, with no deaths and no intensive care admissions for which COVID-19 was the main diagnosis. Other studies have recorded higher rates of critical care admissions; Swann reported that up to 33% of UK neonates with confirmed infection early in the pandemic required critical care,^9^ and a subsequent study over a longer period found that 20% of neonates required critical care.^25^ However, these studies only included admitted babies, and the definition of critical care included admission to a PICU or any level of care in a neonatal unit.^25^ Our data suggest that a much lower proportion of neonates with SARS-CoV-2 infection truly require intensive care.

Despite these positive outcomes, around two-thirds (92/141, 65.2%) of neonates with confirmed SARS-CoV-2 infection had a temporally associated hospital admission. This is perhaps not surprising, as fever is a common sign of SARS-CoV-2 infection in neonates,^1 2 10 20 23 24 28^ and according to UK guidelines,^29^ a temperature above 38°C should prompt blood and urine tests, lumbar puncture and intravenous antibiotics. This demonstrates the indirect effects of SARS-CoV-2 on infants who may receive invasive investigations and treatments aimed at potential bacterial infections. This study was limited in that detailed information on signs and treatments received in hospital was lacking. However, detailed information on the care of babies admitted with SARS-CoV-2 early in the pandemic in the UK is available.^30^

In summary, confirmed SARS-CoV-2 infection in neonates was uncommon over the first 23 months of the pandemic in Scotland. Two-thirds of neonates with confirmed infection had an associated hospital admission. There were no neonatal deaths among babies with confirmed infection. Continued vigilance will be important to assess the ongoing impact of SARS-CoV-2 in the neonatal population as testing, isolation requirements and vaccination programmes continue to evolve, and new viral variants emerge.

## Data Availability

Data may be obtained from a third party and are not publicly available. Patient-level data underlying this article cannot be shared publicly due to data protection and confidentiality requirements. Data access for approved researchers can be authorised by the Public Benefit and Privacy Panel for Health and Social Care (https://www.informationgovernance.scot.nhs.uk/pbpphsc/). Enquiries regarding data availability should be directed to phs.edris@phs.scot.
