# Supplementary material for: Confirmed SARS-CoV-2 infection in Scottish neonates 2020–2022: a national, population-based cohort study
Source: Arch Dis Child Fetal Neonatal Ed. 2023 Jan 6;108(4):367–72. doi: 10.1136/archdischild-2022-324713 (PMC10313998; doi:10.1136/archdischild-2022-324713)
Supplement: Supplementary data [file fetalneonatal-2022-324713supp001.pdf]

Confirmed SARS-CoV-2 infection in Scottish neonates 2020-2022: a population-based cohort study

Supplementary data and figures

Supplementary methods

Study population

We derived and analysed data from the "COVID-19 in Pregnancy in Scotland" (COPS) study dataset as updated in mid-May 2022. The COPS dataset is described in detail elsewhere (1, 2). In brief, it comprises a population-based, dynamic cohort which includes data on all ongoing and completed pregnancies to women in Scotland, and live born babies resulting from those pregnancies, from 1 January 2015 onwards. The dataset is based on linkage of health service and statutory national datasets, including those relating to pregnancy-related care and births, SARS-CoV-2 viral testing and COVID-19 vaccinations, admissions to neonatal and paediatric care, and deaths (1). All individuals in Scotland who receive care from the National Health Service (NHS) (which will include essentially all pregnant women and neonates as there is no private maternity care in Scotland) are assigned a unique patient identifier, the Community Health Index (CHI) number (3) which allows for linkage of medical records relating to an individual. Within the COPS database, the CHI number for both the mother and the baby are included on NHS live birth notification records, further allowing intergenerational linkage of records relating to mothers and their babies. For this study, we included all live born babies born in Scotland between 1st March 2020 and 31st January 2022 who had a valid CHI number available within the COPS dataset.

Identifying confirmed SARS-CoV-2 infections

COPS includes information on all positive SARS-CoV-2 viral tests undertaken on women and babies within the cohort from the NHS Scotland Corporate Data Warehouse. The warehouse includes results of all Reverse Transcription Polymerase Chain Reaction (RT-PCR) SARS-CoV-2 tests processed

through NHS Scotland and UK Government Regional Testing Centre ('Lighthouse') laboratories. It also includes information on all SARS-CoV-2 Lateral Flow Device (LFD) tests where the result has been logged by the individual taking the test (or their parent/carer) on the UK Government website (1, 4). Up to and including 5 Jan 2022, confirmed SARS-CoV-2 infection was defined as a positive viral RT-PCR test result. From 6 Jan 2022 onwards, confirmed infection was defined as a positive viral RT-PCR test result or a positive LFD test result (unless the positive LFD result was followed by a negative viral RT-PCR result within 48 hours). This is consistent with the contemporaneous case definition in Scotland (5).

For any individual, the date that their first positive test sample was taken was used as the date of onset of their first episode of infection. Information on the presence of clinical signs, and date of onset of signs, was not always available on testing records. Subsequent positive samples taken within 90 days of a first positive sample were discounted. A subsequent positive sample taken more than 90 days after a first positive result was taken as indicating a subsequent confirmed infection.

Confirmed neonatal infection was defined as a confirmed infection with date of onset at any point from birth to 27 days old inclusive. By this definition, each infected baby can therefore only have one episode of confirmed infection during the neonatal period. Maternal infection at the time of childbirth was defined as a confirmed infection with date of onset in the 14 days leading to birth, on the day of childbirth, or on the day after giving birth.

For all babies with confirmed neonatal infection, data were obtained from the COPS database regarding: the age of the baby in days at date of onset of infection; maternal age, socioeconomic status, ethnicity, infection status at the time of childbirth; the baby’s sex and gestation at birth. Maternal socioeconomic status was based on the Scottish index of multiple deprivation (SIMD) quintile. SIMD is an area-based measure of material deprivation derived from the postcode of residence (6)).

Identifying admissions of neonates with SARS-CoV-2

Neonates requiring hospital inpatient care may be cared for alongside their mother in a postnatal ward or admitted to a neonatal unit or paediatric ward (including paediatric intensive care units, PICU). Babies who have been discharged home after birth - who then require readmission to hospital -will generally be admitted to a paediatric ward (rather than a neonatal unit) to avoid importing and transmitting infections to neonatal units.

We first identified all admissions to neonatal units and paediatric wards in the neonatal period (date of admission from birth to 27 days old inclusive) for babies in our study population. Admissions to neonatal units were identified through the Scottish Birth Record (SBR, (7)), and admissions to paediatric wards through the Scottish Morbidity Record hospital inpatient and day-case discharge records (SMR01, (8)). A stay in neonatal care was defined as any SBR admission record which included at least one episode in intensive care, high dependency care, or special care. Any SMR01 record in the relevant period was included as a paediatric admission. Of note, this method does not capture neonates who were readmitted from home to a postnatal unit, rather than to a neonatal unit or paediatric care. Therefore, it is possible that some admissions may have been missed.

For babies with confirmed neonatal SARS-CoV-2 infection, we then identified SARS-CoV-2-associated admissions for further analysis. A SARS-CoV-2 associated admission was defined as an admission of a baby with confirmed neonatal infection to neonatal or paediatric care (a) where the date of onset of infection was in the 7 days prior to, or during, the admission (hence date of admission at up to 27+7=34 days old inclusive), or (b) where the admission occurred at any point in the neonatal period (hence date of admission at up to 27 days old inclusive) if COVID-19 was recorded as the main diagnosis (ICD10 code U07.1 or U07.2). An 'admission' was defined as an entire hospital stay from admission to discharge, which may have involved sequential episodes of care in different settings or locations. The main diagnosis recorded for an admission was taken from the diagnostic coding on the first episode of care. Usually, the main diagnosis recorded on an episode record reflects the main condition treated during that episode, however from the start of the pandemic to May 2021 inclusive, a temporary amendment to national coding guidance advised that COVID-19 (ICD10 code

U07.1 or U07.2) should always be coded as the main diagnosis if present during an episode of care (9).

Neonatal and paediatric admission records for these SARS-CoV-2-associated admissions were analysed to identify the highest level of care provided in the neonatal unit (intensive care, high dependency, or special care) or whether the admission included an episode in a paediatric intensive care unit, length of stay for the entire admission, whether COVID-19 was recorded as the main diagnosis, and whether the infection was likely to be nosocomial. A probable nosocomial infection was defined as when the first positive test was taken on day 7 of an ongoing admission or later.

Calculation of rates and confidence intervals

Due to the relatively small numbers involved, all data reported here are descriptive only and no formal statistical comparisons have been made. Rates were calculated using the number of babies with confirmed neonatal infection (numerator) and the total number of live births (denominator) during the relevant time period (individual months or the full study period 1 March 2020 to 31 January 2022), with confidence intervals calculated using Wilson score estimates. This approach to calculating rates supports production of the timeliest results, however it does allow a mismatch between numerator and denominator. For example, in any one month some of the babies with confirmed infection during that month may have been born in the previous month. Given the number of live births is fairly consistent month to month, we believe that this mismatch should have minimal impact on the interpretation of our findings.

Table S1

Monthly rate of confirmed SARS-CoV-2 infection in babies aged 27 days and under per 100,000 live births, Scotland 1 March 2020 to 31 January 2022.

**Month**	**Total number of live births**	**Number of confirmed SARS-CoV-2 infections in neonates with date of onset in month**	**Overall rate of confirmed SARS-CoV-2 infection in neonates (per 100,000 live births)**	**Lower CI**	**Upper CI**
**Mar-20**	4,001	0	0.0	0.0	119.6
**Apr-20**	3,857	2	51.9	9.0	208.9
**May-20**	3,888	0	0.0	0.0	123.1
**Jun-20**	4,083	0	0.0	0.0	117.2
**Jul-20**	4,282	0	0.0	0.0	111.8
**Aug-20**	4,078	2	49.0	8.5	197.6
**Sep-20**	4,106	1	24.4	1.3	158.0
**Oct-20**	4,152	5	120.4	44.3	298.4
**Nov-20**	3,703	2	54.0	9.4	217.6
**Dec-20**	3,707	4	107.9	34.6	296.4
**Jan-21**	3,641	9	247.2	120.7	487.1
**Feb-21**	3,447	2	58.0	10.1	233.7
**Mar-21**	3,966	2	50.4	8.7	203.2
**Apr-21**	3,742	3	80.2	20.7	255.2
**May-21**	4,022	3	74.6	19.3	237.4
**Jun-21**	4,006	8	199.7	92.9	410.1
**Jul-21**	4,445	3	67.5	17.4	214.9

**Aug-21**	4,342	18	414.6	253.5	668.5
**Sep-21**	4,339	16	368.8	218.3	612.4
**Oct-21**	4,308	14	325.0	185.0	559.3
**Nov-21**	4,048	10	247.0	125.6	470.1
**Dec-21**	3,937	11	279.4	147.0	515.9
**Jan-22**	3,909	26	665.1	443.9	988.0
**Total**	92,009	141	153.3	129.5	181.3

CI = 95% confidence interval.

Table S2

Rates of confirmed SARS-CoV-2 infection in post neonatal infants and children up to 17 years of age, Scotland 1 March 2020 to 31 January 2022. Estimated number of children in each age group (denominator for rates) taken from National Records of Scotland, Mid-2020 Population Estimates Scotland and is kept constant throughout (10). Number of post neonatal infants (under 1 year) estimated using the Mid-2020 Population Estimate minus 4,000 (the average number of live births per month). Number of positive tests obtained from NHS Scotland Corporate Data Warehouse (4).

Month	Estimated no. of post-neonates, under 1yr (d28 to d364)	No. confirmed SARS-CoV-2 infection post-neonates	Overall rate post-neonates (per 100,000)	Estimated no. of children aged 1-4 years	No. confirmed SARS-CoV-2 infection aged 1-4 years	Overall rate aged 1-4 years (per 100,000)	Estimated no. of children aged 5-11 years	No. confirmed SARS-CoV-2 infection aged 5-11 years	Overall rate children aged 5-11 years (per 100,000)	Estimated no. of children aged 12-17 years	No. confirmed SARS-CoV-2 infection children aged 12-17 years	Overall rate of children aged 12-17 years (per 100,000)
**Mar-20**	44,646	8	17.9	215,171	8	3.7	418,842	8	1.9	344,274	12	3.5
**Apr-20**	44,646	1	2.2	215,171	14	6.5	418,842	23	5.5	344,274	39	11.3
**May-20**	44,646	1	2.2	215,171	4	1.9	418,842	27	6.6	344,274	44	12.8
**Jun-20**	44,646	0	0.0	215,171	3	1.4	418,842	5	1.2	344,274	11	3.2
**Jul-20**	44,646	0	0.0	215,171	2	0.9	418,842	7	1.7	344,274	2	0.6
**Aug-20**	44,646	10	22.4	215,171	27	12.6	418,842	55	13.1	344,274	115	33.4
**Sep-20**	44,646	11	24.6	215,171	67	31.1	418,842	178	42.5	344,274	550	159.8
**Oct-20**	44,646	71	159.0	215,171	323	150.1	418,842	828	197.7	344,274	1,580	458.9
**Nov-20**	44,646	77	172.5	215,171	363	168.7	418,842	1,164	277.9	344,274	1,903	552.8
**Dec-20**	44,646	101	226.2	215,171	420	195.2	418,842	1,176	280.8	344,274	1,733	503.4
**Jan-21**	44,646	154	344.9	215,171	599	278.4	418,842	1,322	315.6	344,274	1,977	574.3
**Feb-21**	44,646	89	199.4	215,171	524	243.5	418,842	892	213.0	344,274	970	281.8

**Mar-21**	44,646	67	150.1	215,171	768	356.9	418,842	1,932	461.3	344,274	1,038	301.5
**Apr-21**	44,646	41	91.8	215,171	296	137.6	418,842	755	180.3	344,274	645	187.4
**May-21**	44,646	44	98.6	215,171	363	168.7	418,842	1,465	349.8	344,274	1,135	329.7
**Jun-21**	44,646	162	362.9	215,171	1,039	482.9	418,842	4,602	1098.7	344,274	5,003	1453.2
**Jul-21**	44,646	242	542.0	215,171	1,225	569.3	418,842	4,128	985.6	344,274	7,011	2036.5
**Aug-21**	44,646	258	577.9	215,171	1,720	799.4	418,842	10,034	2395.7	344,274	14,200	4124.6
**Sep-21**	44,646	380	851.1	215,171	2,470	1147.9	418,842	19,740	4713.0	344,274	22,843	6635.1
**Oct-21**	44,646	247	553.2	215,171	1,470	683.2	418,842	11,279	2692.9	344,274	8,054	2339.4
**Nov-21**	44,646	277	620.4	215,171	2,149	998.7	418,842	16,748	3998.6	344,274	8,311	2414.1
**Dec-21**	44,646	615	1377.5	215,171	3,753	1744.2	418,842	20,901	4990.2	344,274	16,654	4837.4
**Jan-22**	44,646	1,071	2398.9	215,171	8,182	3802.6	418,842	35,480	8471.0	344,274	21,149	6143.1
**Total**	44,646	3,927	8795.9	215,171	25,789	11985.4	418,842	132,749	31694.3	344,274	114,979	33397.5

Table S3:

Rates of confirmed SARS-CoV-2 infection in pregnancy, Scotland 1 March 2020 to 31 January 2022. Data obtained from COPS cohort.

Month	Estimated No. women in Scotland with an ongoing pregnancy at start of month*	No. women with confirmed SARS-CoV-2 infection in pregnancy with date of onset in month**	Rate per 100,000 pregnant women***
**Mar-20**	38,018	18	47.4
**Apr-20**	37,557	35	93.2
**May-20**	37,017	23	62.1
**Jun-20**	36,698	2	5.5
**Jul-20**	36,536	3	8.2
**Aug-20**	36,183	14	38.7
**Sep-20**	36,086	61	169.0
**Oct-20**	36,003	286	794.4
**Nov-20**	36,254	261	719.9
**Dec-20**	36,837	324	879.6
**Jan-21**	37,647	476	1264.4
**Feb-21**	38,311	235	613.4
**Mar-21**	38,773	189	487.5
**Apr-21**	38,490	90	233.8
**May-21**	38,626	145	375.4
**Jun-21**	38,491	488	1267.8
**Jul-21**	38,363	796	2074.9
**Aug-21**	37,893	820	2164.0
**Sep-21**	37,229	975	2618.9
**Oct-21**	36,708	634	1727.1

**Nov-21**	36,131	688	1904.2
**Dec-21**	36,169	2,436	6735.1
**Jan-22**	36,514	2,986	8177.7
**Total**	151,230	10,552	6,977.5

* The total for this column is the number of women who were pregnant at some point during the study period.

** The total for this column is the number of women with confirmed SARS-CoV-2 infection in pregnancy during the study period.

*** The total for this column is the overall rate of confirmed SARS-CoV-2 infection in pregnancy during the study period.

Table S4

Date of onset of first positive test for neonates who had a confirmed SARS-CoV-2 infection at or below 27 days of age, Scotland 1 March 2020 to 31 January 2022.

Date of onset of infection	Number of confirmed SARS-CoV-2 infection in neonates	Cumulative number of confirmed SARS-CoV-2 infection in neonates
d0 (date of birth)	2	2
d1	2	4
d2	2	6
d3	4	10
d4	1	11
d5	7	18
d6	1	19
d7	3	22
d8	3	25
d9	4	29
d10	8	37
d11	6	43
d12	6	49
d13	4	53
d14	5	58
d15	4	62
d16	8	70
d17	8	78
d18	7	85
d19	7	92
d20	5	97

d21	7	104
d22	3	107
d23	5	112
d24	4	116
d25	8	124
d26	9	133
d27	8	141

Table S5

Admission rates for neonates with confirmed SARS-CoV-2 infection, Scotland 1 March 2020 to 31 January 2022. Rounded to 1 decimal place.

Month	Total number of live births	No. neonates with confirmed SARS-CoV-2 infection	No with associated hospital admission	No with associated admission with COVID-19 as main diagnosis	Overall rate of confirmed SARS-CoV-2 infection in neonates (per 100,000 live births)	Rate of confirmed neonatal infection with associated admission (per 100,000 live births)	Rate of confirmed neonatal infection with associated admission with COVID-19 as main diagnosis (per 100,000 live births)
**Mar-20**	4,001	0	0	0	0.0	0.0	0.0
**Apr-20**	3,857	2	2	0	51.9	51.9	0.0
**May-20**	3,888	0	0	0	0.0	0.0	0.0
**Jun-20**	4,083	0	0	0	0.0	0.0	0.0
**Jul-20**	4,282	0	0	0	0.0	0.0	0.0
**Aug-20**	4,078	2	0	0	49.0	0.0	0.0
**Sep-20**	4,106	1	1	1	24.4	24.4	24.4
**Oct-20**	4,152	5	1	1	120.4	24.1	24.1

**Nov-20**	3,703	2	2	2	54.0	54.0	54.0
**Dec-20**	3,707	4	1	1	107.9	27.0	27.0
**Jan-21**	3,641	9	3	3	247.2	82.4	82.4
**Feb-21**	3,447	2	0	0	58.0	0.0	0.0
**Mar-21**	3,966	2	2	0	50.4	50.4	0.0
**Apr-21**	3,742	3	1	0	80.2	26.7	0.0
**May-21**	4,022	3	2	2	74.6	49.7	49.7
**Jun-21**	4,006	8	6	4	199.7	149.8	99.9
**Jul-21**	4,445	3	3	2	67.5	67.5	45.0
**Aug-21**	4,342	18	13	8	414.6	299.4	184.3
**Sep-21**	4,339	16	13	11	368.8	299.6	253.5
**Oct-21**	4,308	14	7	5	325.0	162.5	116.1
**Nov-21**	4,048	10	8	6	247.0	197.6	148.2
**Dec-21**	3,937	11	6	2	279.4	152.4	50.8
**Jan-22**	3,909	26	21	12	665.1	537.2	307.0
**Total**	92,009	141	92	60	153.3	100.0	65.2

Figure S1

Trends in overall rate of confirmed neonatal SARS-CoV-2 infection compared to rate of neonatal infection with associated hospital admission, and with associated admission with COVID-19 coded as main diagnosis, Scotland 1 March 2020 to 31 January 2022. Per 100,000 live births.

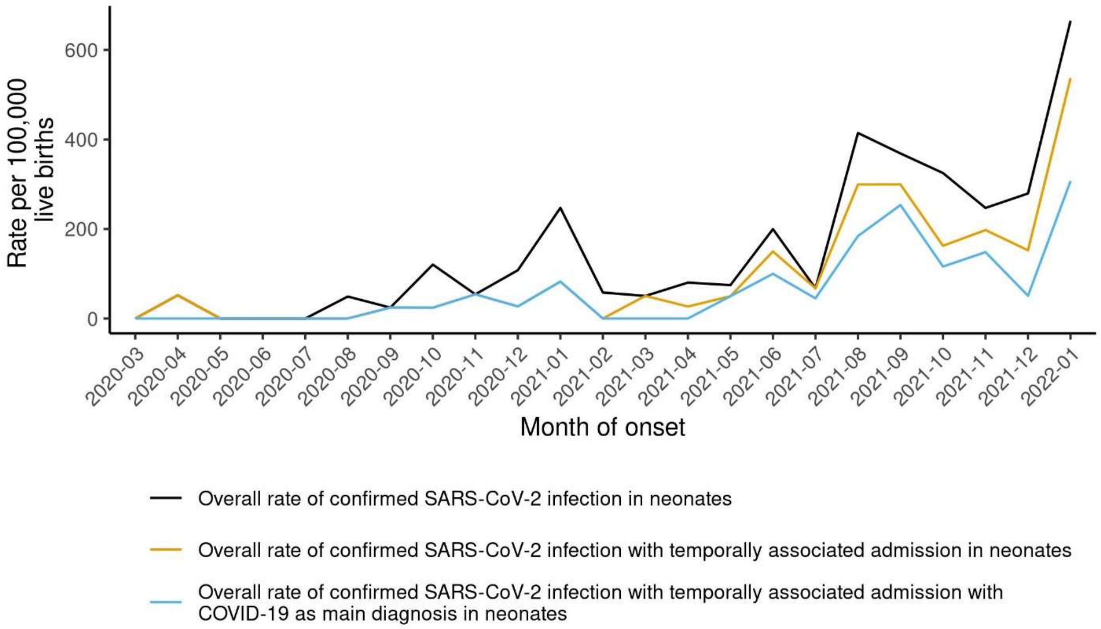

Additional references

1. Stock SJ, Carruthers J, Denny C, et al. Cohort Profile: The COVID-19 in Pregnancy in Scotland (COPS) dynamic cohort of pregnant women to assess effects of viral and vaccine exposures on pregnancy. Int J Epidemiol. 2022.

2. Stock SJ, McAllister D, Vasileiou E, et al. COVID-19 in Pregnancy in Scotland (COPS): protocol for an observational study using linked Scottish national data. BMJ Open. 2020;10(11):e042813.

3. Information Services Division Scotland. CHI: ISD Scotland Data Dictionary 2022 [Available from: https://www.ndc.scot.nhs.uk/Dictionary-A-Z/Definitions/index.asp?ID=128&Title=CHI%20Number.

4. Public Health Scotland. COVID-19 Daily dashboard: Notes about the data 2022 [Available from: https://publichealthscotland.scot/media/12327/coviddailydashboardnotes.pdf.

5. Public Health Scotland . COVID-19 - guidance for health protection teams (HPTs). 2022.

6. Scottish Government. Introducing the Scottish Index of Multiple Deprivation 2020. National statistics publication; 2020.

7. Information Services Division Scotland. Scottish Birth Record : ISD Scotland Data Dictionary . 2022 [Available from: https://www.ndc.scot.nhs.uk/Data-Dictionary/SMR-Datasets/Scottish-Birth-Record/.

8. Information Services Division Scotland. SMR01: ISD Data dictionary 2022 [Available from: https://www.ndc.scot.nhs.uk/Dictionary-A-Z/Definitions/index.asp?Search=S&ID=460&Title=SMR01%20-%20General/Acute%20Inpatient%20and%20Day%20Case.

9. Public Health Scotland. Scottish Clinical Coding Standards Number 29. 2021. Available from https://www.isdscotland.org/Products-and-Services/Terminology-Services/Clinical-Coding-Guidelines/Docs/Scottish-clinical-coding-standards-no29-July-2021.pdf. Last accessed 28th October 2022.

10. National Records of Scotland. Mid-2020 Population Estimates Scotland. 2021.
